# Strategies for Bacterial Eradication from Human and Animal Semen Samples: Current Options and Future Alternatives

**DOI:** 10.3390/s23156978

**Published:** 2023-08-06

**Authors:** Michal Ďuračka, Filip Benko, Milan Chňapek, Eva Tvrdá

**Affiliations:** 1 AgroBioTech Research Centre, Slovak University of Agriculture in Nitra, Tr. A. Hlinku 2, 949 76 Nitra, Slovakia; michal.duracka@uniag.sk; 2 Institute of Applied Biology, Faculty of Biotechnology and Food Sciences, Slovak University of Agriculture in Nitra, Tr. A. Hlinku 2, 949 76 Nitra, Slovakia; xbenkof@uniag.sk; 3 Institute of Biotechnology, Faculty of Biotechnology and Food Sciences, Slovak University of Agriculture in Nitra, Tr. A. Hlinku 2, 949 76 Nitra, Slovakia; milan.chnapek@uniag.sk

**Keywords:** bacteriospermia, antibiotics, sperm selection methods, alternatives

## Abstract

The primary role of semen processing and preservation is to maintain a high proportion of structurally and functionally competent and mature spermatozoa, that may be used for the purposes of artificial reproduction when needed, whilst minimizing any potential causes of sperm deterioration during ex vivo semen handling. Out of a multitude of variables determining the success of sperm preservation, bacterial contamination has been acknowledged with an increased interest because of its often unpredictable and complex effects on semen quality. Whilst antibiotics are usually the most straight-forward option to prevent the bacterial contamination of semen, antimicrobial resistance has become a serious threat requiring widespread attention. As such, besides discussing the consequences of bacteriospermia on the sperm vitality and the risks of antibiotic overuse in andrology, this paper summarizes the currently available evidence on alternative strategies to prevent bacterial contamination of semen prior to, during, and following sperm processing, selection, and preservation. Alternative antibacterial supplements are reviewed, and emphasis is given to modern methods of sperm selection that may be combined by the physical removal of bacteria prior to sperm preservation or by use in assisted reproductive technologies.

## 1. Introduction

Early attempts for sperm vitality conservation may be traced back to 1776, when Spallanzani froze spermatozoa using snow and subsequently demonstrated their motility after rewarming [1]. Since then, remarkable progress in the evolution of techniques and media for short- or long-term semen storage has allowed sperm preservation to overcome numerous space and time limitations, and thus to become an integral part of assisted reproduction technologies (ARTs) in animals and humans [1,2].

Sperm preservation, at low temperatures and when properly performed, enables the long-term storage of male gametes in a state of metabolic arrest that prevents cellular senescence while maintaining their viability and fertilizing potential, therefore this allows them to be used when and where they are needed [2]. While the role of sperm storage at low temperatures as a critical pillar of reproductive technologies is undoubtable, the rationale behind its use for the management of male fertility may differ between animal and human ARTs [3,4,5,6,7]. In humans, sperm storage represents an effective strategy to preserve male reproductive capacity following cytotoxic or surgical treatment that may cause possible testicular or ejaculatory dysfunction. Cryopreservation may also be routinely used in men who are exposed to potentially toxic agents which may interfere with spermatogenesis, or who begin assisted reproduction treatment, and thus have a back-up sperm source [3,4]. In donor insemination programs, sperm preservation is necessary to have enough time to screen the donors for possible exclusion criteria, before the semen sample is used for clinical purposes [4]. In animals, sperm preservation techniques are extensively employed to manage or accelerate the rate of genetic improvement, by using a lower number of studs with valuable traits to inseminate a large number of females, leading to a significant reduction in the cost of the breeding process [5,6]. Semen storage is also a valuable tool for the protection of genetic resources and biodiversity of protected or endangered animal species [7].

Although an important benefit of artificial insemination (AI) lies in the prevention of disease transmission, potential pathogens may be easily disseminated through an often-overlooked contaminated semen [8]. The term “bacteriospermia” refers to the presence of bacteria in the ejaculate [9] and is regarded as clinically relevant when the bacterial count exceeds 1000 colony-forming units (CFU)/mL semen [10]. In most cases, the condition is often associated with an acute or chronic bacterial infection of one or more organs of the male urogenital system, including the testes, epididymides, prostate, ureter, or urethra [8]. Gram-positive, Gram-negative, as well as Gram-unstainable bacteria may be regarded as causative agents for bacteriospermia associated with urogenital infection, out of which *Escherichia coli*, *Staphylococcus aureus*, *Ureaplasma urealyticum*, *Chlamydia trachomatis*, *Mycoplasma genitalium*, *Enterococcus faecalis*, and streptococci have received the most attention [10,11]. At the same time, large quantities of bacteria may be present even in semen samples collected from otherwise healthy and fertile subjects [12,13,14,15,16,17], since the male urogenital system naturally houses an inherent bacteriome [18,19]. Semen may also become contaminated by bacteria present in the prepuce and penile foreskin during ejaculation [20]. The bacterial contamination of particularly animal semen may also stem from skin, wool, bedding, or excretions [21]. Moreover, semen collection and processing procedures may not be per se carried out in an antiseptic environment, thus ejaculates may be infested by bacteria proceeding from electroejaculators or artificial vaginas, laboratory glassware, contaminated semen extenders and ART media, or poor on-site hygiene standards [21,22].

Under ideal circumstances, semen extenders provide protection to male gametes and preserve their motion and fertilizing ability over time, primarily by stabilizing the plasma membrane, providing sources of energy, and preventing any harmful effects of pH and osmolarity fluctuations or oxidative stress on spermatozoa [23]. On the other hand, sperm preservation media may act as a rich reservoir of nutrients and a favorable environment for bacterial growth and multiplication if a contaminated ejaculate is processed for ART or AI [24]. Although bacterial growth largely depends on the temperature, some bacteria are capable of growing, to some extent, even during cooling and/or freezing [25]. What is more, bacteria may survive even in frozen semen samples, resuming growth shortly following thawing [24]. In this sense, the time necessary for semen processing, dilution, cooling, freezing, and thawing should be kept to a minimum and strict hygiene measures should always be followed.

While the possibility of the initial bacterial contamination of semen is currently not taken into consideration by the current guidelines, neither for human nor for animal ejaculates [26], a vast collection of evidence indicates that bacteriospermia may lead to depleted sperm motion activity and membrane integrity [12,13,14,15,16,17,27,28], morphological aberrations to the sperm head, acrosome, mid-piece, and tail [10,13,14,27,28,29,30], a deteriorated mitochondrial function and ATP production [13,14,15,16,17,30], DNA fragmentation, and the onset of cell death [13,14,15,16,17,27,28,29,30]. Sperm agglutination [28,30,31], the release of bacterial toxins and cytotoxic cytokines, as well as high levels of reactive oxygen species (ROS) have also been frequently reported in ejaculates compromising bacteria [13,14,15,16,17,27,31]. It has also been revealed that the processing of a contaminated semen sample could modify the biochemical properties of semen extenders or ART media, which may compromise the shelf life of extended semen [22,26,32]. Subsequently, the use of bacteriospermic samples for AI may lead to the onset of various infections in the female [33,34,35] and contribute to lower embryo survival and pregnancy outcomes [36,37].

Based on the severity of consequences bacteriospermia may cause in the field of andrology and reproductive biology, this review discusses the strategies employed to prevent or manage the spread and persistence of bacteria in ejaculates, starting from conventional and currently employed approaches to alternative options with a promising perspective for the future.

## 2. Antibiotics

Antibiotics are currently the most preferred option for bacterial control during semen processing and storage, primarily because of their affordability, diversity, biological activity, and availability [38].

In the case of bovine and porcine ejaculates, Directives 88/407 and 90/429 of the European Council, Annex C2 state that an effective combination of antimicrobials, especially against mycoplasmas and leptospires, should be supplemented to each extended semen sample [39,40]. This combination must lead to an effect equivalent to 500 µg streptomycin/mL final dilution; 500 IU penicillin/mL final dilution; 150 µg lincomycin/mL final dilution; and/or 300 µg spectinomycin/mL final dilution. This primary legislative document was later supported by the Directive 92/65 [41], which is relevant to other animal species such as small ruminants, poultry, and *Equidae*, and which reports another combination of antimicrobials consisting of 50 μg tylosin/mL, 250 μg gentamicin/mL, and 150/300 μg lincomycin/spectinomycin/mL; or 25 μg divecacin/mL and 75 μg amikacin/mL, which may be used alone or in combination [41]. A similar approach is usually followed in human andrology, although no specific guidelines have been published by the World Health Organization (WHO) laboratory manual for the examination and processing of human semen [42].

The antibiotic substances that are most frequently used in semen diluents include β-lactams (penicillins, cephalosporins), whose mechanism of action lies in the inhibition of the bacterial cell wall synthesis, leading to cell lysis and death. Others include inhibitors of bacterial protein synthesis such as aminoglycosides (streptomycin, gentamicin, kanamycin), lincosamides (lincomycin) or macrolides (tylosin, tilmicosin, spectinomycin). Nevertheless, a large variation exists among different species with respect to the antimicrobial types and doses added to extenders used for both semen chilling and freezing procedures [43]. Furthermore, the exact combination and/or concentration of antibiotics in commercially available semen diluents and ART media is generally undisclosed by the producer.

Nevertheless, a convincing body of evidence gathered from recent studies emphasizes the presence of diverse bacterial strains found in native semen, insemination doses, ART media with concerning resistance patterns to antibiotics routinely used in andrology, animal production, and reproductive medicine (Table 1). These recurrent patterns may be accompanied by the horizontal or vertical transmission of bacterial resistance, which may pose a concerning threat to public health, the food chain, and the stability of ecosystems [44]. Furthermore, several studies have reported the significant negative effect of antibiotic supplements, such as gentamicin, streptomycin-penicillin, ciprofloxacin or doxycycline, on sperm motility, membrane, and DNA integrity as well as overall fertilization ability, stressing the need for the prudent use of antibiotics during sperm preservation [45,46,47]. Thus, the search for alternative options that would eliminate bacteria from semen by different means, while preventing the spread of bacterial resistance and additional cytotoxicity, has become a reality. Specific approaches will be discussed in detail in the following sections.

## 3. Antimicrobial Proteins, Biocompounds, Plant Extracts, and Nanoparticles

More than 5500 antimicrobial peptides (AMPs) are known for their effects against pathogens. Despite their wide structural diversity, most AMPs contain polar and non-polar parts in their structure [61]. Although the mechanism of action of AMPs is only partially investigated, most of these have studied bacteriostatic peptides belonging to the class of cationic amphipathic helices [62]. The advantage of this class is that these peptides show favorable selectivity against microbes when compared to hydrophobic and anionic peptides. Magainin is the most studied cationic peptide, consisting of 23 amino acids, and is currently used as a therapeutic [63]. Only a few studies reported the use of AMPs in semen storage. Hensel et al. [64] tested the effect of seven short cationic lipopeptides on porcine spermatozoa. Two lipopeptides did not show significant impairment of sperm quality while being antibacterial against several bacteria including *Alcaligenes faecalis*, *Escherichia coli*, *Pseudomonas aeruginosa*, *Treuperella pyogenes*, *Pasteurella* sp., *Providencia stuartii*, and *Streptococcus porcinus*. The membrane selectivity of AMPs lies in two specific factors: the first—an electrostatic interaction between the cationic peptide and high-acid bacterial membrane, and the second—high cholesterol amount in eucaryotic membranes stabilizes and makes them more rigid when compared to procaryotic membranes [65]. According to Bussalleu et al. [66], PMAP-37 at 3 µM did not affect boar spermatozoa quality while being effective against bacteria. The advantages of AMPs lie in their thermostability, economic availability, and ability to minimize the risk of antimicrobial resistance emergence [67]. On the other hand, there are several reasons why the use of naturally obtained AMPs is limited: susceptibility to proteases, cytotoxicity to host cells, inactivity at physiological salt concentrations, and sustainability of production. Therefore, the focus has shifted to the development of synthetic AMPs [68]. As AMPs and conventional antibiotics synergize antimicrobial activity, future strategies should deal with their mutual use. The combination of AMPs and antibiotic supplementation could reduce the minimal inhibitory concentration and cell toxicity.

Numerous studies have already pointed out that bioactive compounds may improve the oxidative milieu of stored semen, thus prolonging sperm survival in human [69], boar [70], bull [71], ram [72], and rooster semen [73]. In particular, flavonoids are the most important class of phenolic compounds exerting extraordinary antibacterial activity against multi-drug resistant bacterial strains [74]. Several mechanisms are behind their antibacterial effect, including the suppression of nucleic acid synthesis, membrane function, and energetic metabolism [75]. Moreover, bacterial adhesion, the formation of porins, and biofilm formation were reduced in the presence of flavonoids [76]. As previously noticed, the use of bioactive substances as antimicrobial supplements in semen storage is limited by their cytotoxic effects at high concentrations. The combination of carvacrol and thymol was found to be effective against present bacterial strains in stored boar semen (17 °C) without any significant effect on sperm motility and viability [77]. Amongst the flavonoids, 15 mg/L of icariin showed both antioxidant and antimicrobial effects in stored (17 °C) boar semen, while a combination with 62.5 mg/L of gentamicin instead of 250 mg/L showed similar effects on bacterial inhibition [78]. In the meantime, curcumin at a concentration of 5% significantly reduced the number of bacterial colonies within 2 h in stored bull semen and did not negatively influence spermatozoa.

Moreover, plant extracts also showed favorable effects on stored semen [79]; similarly to pure bioactive substances, their antibacterial effect in the storage medium could be as effective as sperm resistance to their cytotoxic effect at high concentrations. *Schizandra chinensis* extract presented with a minimal inhibitory concentration at 64.2 µg/mL which was effective against *Enterococcus faecalis* and *Listeria monocytogenes* and still did not negatively affect the functional activity and structural integrity of stored bull spermatozoa. However, most of the studied bacteria were susceptible to concentrations higher than 75 µg/mL, which could act as a prooxidant. Rosemary was previously reported to have an antibacterial effect in the form of essential oil in stored boar semen [80]. On the other hand, rosmarinic acid did not reveal any antibacterial activity against bacteria in bull semen, which indicates that the antibacterial activity of supplements stemming from plant sources may vary depending on their form.

In recent years, nanoparticles were found to be interesting antibacterial agents. However, only a few studies have been published concerning semen storage. A slight antibiotic effect with no detrimental effects on the sperm quality was observed when iron oxide nanoparticles were added [81]. The desired antibacterial effect was recorded when iron and silver nanoparticles were combined at the cost of increased spermatotoxicity [82]. Yousef et al. [83] disclosed that concentrations ≤30 µg/mL of silver-carbon nanoparticles did not affect sperm quality parameters during bull semen storage and were effective against *Staphylococcus aureus*, *Pseudomonas aeruginosa*, and *Escherichia coli*, although transmission electron microscopy revealed that these nanoparticles were attached to the plasma membrane but did not penetrate the bull sperm cell. However, it is not known how the nanoparticles would affect the recipients’ genitalia and embryo production. Finally, chitosan-nanoparticles, combined with ethylenediaminetetraacetic acid (EDTA) and bestatin, provided a bacteriostatic effect against *Enterobacteriaceae* without damaging rabbit spermatozoa stored at 15 °C for 72 h [84].

## 4. Centrifugation Techniques

Repeated washing and centrifuging of a large volume of semen in a culture medium represents the simplest removal technique of bacteria present in the seminal fluid. Usually, 2–3 centrifugations (not greater than 800× *g*) after mixing with 5–10× of culture medium are recommended [85]. The disadvantage of this straightforward method is that all sperm cells are present in the pellet including abnormal, immature, and dead spermatozoa as well as leukocytes and epithelial cells. Such unsorted samples could inhibit the capacitation process and increase the development of antisperm antibodies following insemination [86]. Moreover, oxidative damage to the sperm chromatin was reported, and since then the simple centrifugation method was no longer recommended [87].

The swim-up technique belongs to procedures where the active motion of spermatozoa plays a key role in the separation of highly motile sperm. The nutritional support is provided by antioxidants present in the culture medium. For human semen, the sample (1 mL) is gently prewashed, placed at the bottom of a 15 mL conical test tube, and covered by the sperm wash medium (1.2 mL). The tube is incubated for 60 min at an angle of 45°. The upper supernatant is sterilely collected [42]. Several modifications of swim-up technique were also reported for humans [88,89,90,91] and animal species [92,93,94]. Chen et al. [95] reported that 57.4% of semen samples processed by swim-up were bacteria-free, and only 23.8% were retrieved in the case of density gradient centrifugation. However, the most reliable centrifugation technique to date is density gradient centrifugation with subsequent swim-up. Almost 98% of the semen samples were reported to be free from bacteria.

Density gradient centrifugation is well-known in semen processing laboratories since it is a relatively simple method used in different cell separations based on density. The density of morphologically normal human sperm is 1.10 g/mL while, in the case of morphologically abnormal and immature sperm, the density is 1.06–1.09 g/mL [96]. Lindahl and Kihlström [97] reported normal bull sperm density varying between 1.241 and 1.334 g/mL. On the other hand, Lavon et al. [98] reported a range from 1.0376 to 1.0927 g/mL, because the density of sperm may change with age [99]. The basis of this separation type is a colloidal medium formed by silica particles coated with covalently bonded hydrophilic silane in HEPES [100]. Usually, two gradients are used to obtain morphologically normal motile spermatozoa with intact membranes: a denser colloid is placed at the bottom of a falcon tube and a sparser colloid is gently layered on top of the denser colloid without mixing. The common concentration and volume ratio between these phases is 1:1. The semen sample is carefully layered on top of the sparser phase and centrifuged. During centrifugation, highly motile spermatozoa reach the bottom easier than slowly moving or immotile sperm cells. After centrifugation, interphases seminal plasma/sparser colloid and sparser/denser colloid contain abnormal cells, cell debris, leukocytes, and immotile or poor motility spermatozoa. Most of the bacteria stay in the seminal plasma on top of the falcon tube. The pellet is formed by the most viable spermatozoa. Therefore, it is necessary to aseptically remove the supernatant. The pellet is washed, centrifuged, and resuspended with a washing medium again. The centrifugal force should be kept under 300× *g* to minimize ROS production [101,102]. To keep aseptic conditions, Nicholson et al. [102] strictly recommend changing Pasteur pipettes and tubes before washing. According to their results, approximately 30 colonies per 10 mL of semen were detected in fresh ejaculates obtained from men undergoing fertility investigation, while 3 colonies were counted after density gradient centrifugation, and less than 1 colony when pipettes and tubes were changed before washing. Single-layer centrifugation represents a modified density gradient separation when only one phase of colloid is used to separate viable spermatozoa [103]. Both single- and double-layer centrifugation can effectively improve the quality of collected semen [104]. However, the comparison of the effectivity of both methods is unknown when it comes to the elimination of bacteria. Morrell et al. [105] reported the removal of more than 90% of the bacterial load of >5 × 10^4^ CFU/mL thanks to colloid centrifugation. What is more, higher removal effectivity was observed with lower bacterial loads. So far, human [106], bovine [103], boar [107], and equine [108] semen was successfully processed through colloid centrifugation to eliminate bacteria from samples. Despite the fact that several studies reported an improvement in the proportion of non-fragmented spermatozoa [109,110], there is probably still a certain degree of iatrogenic DNA damage because of centrifugation [111].

## 5. Non-Thermal Plasma

Plasma, regarded as the fourth fundamental state of matter, is defined as a quasi-neutral system in a gaseous or fluid-like form which can be artificially created in an electromagnetic field coupled with a flow of neutral gases such as oxygen, argon, helium, nitrogen, or atmospheric air [112,113]. Plasma is a heterogenous mixture composed of reactive oxygen species (hydrogen peroxide, ozone, etc.), reactive nitrogen species (RNS; nitrogen dioxide, nitric oxide, etc.), charged particles, excited atoms, electrical field, positive and negative ions, and UV radiation [114,115,116], all of which are known to possess strong antimicrobial properties. Their synergistic action results in the numerous benefits of plasma as an efficient, cost-effective, and non-toxic bactericidal agent [112].

Two plasmas exist based on the temperature and relative energy levels of electrons and particles, specifically high-temperature (thermal or fusion) plasmas and low-temperature (non-thermal or cold) plasmas [117]. As opposed to thermal plasma, non-thermal plasma (NTP) is generated at low or atmospheric pressure, containing heavy particles at room temperature and electrons at higher temperature [117,118]. Consequently, NTP application does not produce the high heat that would cause potential side-effects or harm when applied to living tissues, cells, or biomaterials [117,119].

**Figure 1:**
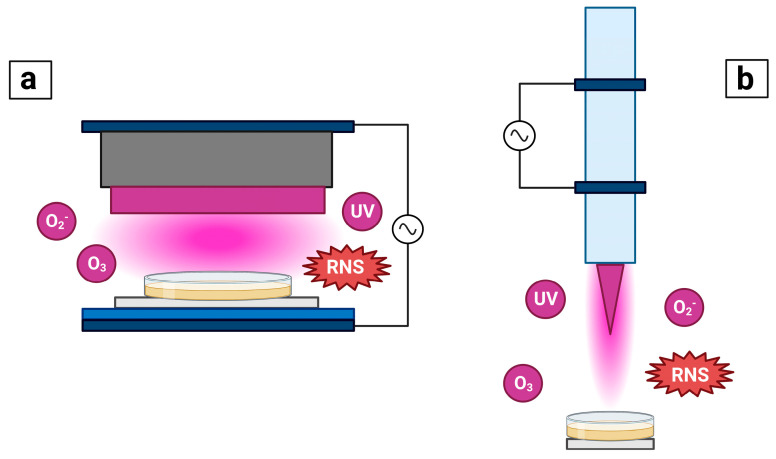
Most common non−thermal plasma configurations: (**a**) dielectric barrier discharge (DBD) plasma and (**b**) plasma jet.

The two most commonly used NTP configurations are the dielectric barrier discharge (DBD) and plasma jet [113] (Figure 1). The principle of DBD lies in an electrical discharge between two electrodes at atmospheric pressure and air where one electrode is supplied with a high voltage, while the other one is grounded. The high voltage electrode is enclosed within a dielectric material, such as glass or silica glass, ceramics, polymers, or quartz, whose role is to limit and/or block the discharge current [120]. Depending on the plasma configuration, the gas employed or the voltage of the DBD model, the distance between electrodes may range from micrometers to centimeters while the operating frequencies oscillate around the tens of kHz [121]. In general, DBD plasma takes advantage of atmospheric air as the working gas [113]. On the other hand, a plasma jet is a tube with electrodes placed either inside or around it, through which gas flows and ionizes once it passes through the electrical field created between the electrodes [122,123]. This plasma configuration may use a variety of working gases such as oxygen, argon, nitrogen, or helium. Unlike an arc created by the DBD plasma, jet plasma is ignited inside the tube and transported outside in a thin jet stream [113,122,123].

Depending on the objective and the plasma configuration use, three basic approaches may be used to treat samples with NTP: (1) Direct plasma treatment, which uses the target area as a counter electrode leading to homogenous plasma production with high levels of generated plasma components (DBD plasma) [124]. (2) Indirect plasma treatment, in which the plasma itself is generated between two electrodes and subsequently transported to the target area either by diffusion or a carrier gas (plasma jets) [113,124]. (3) Hybrid plasma treatment, where plasma is produced in multiple discharges such as a grounded wire mesh electrode (surface micro discharge) [113].

Non-thermal plasma has emerged as an alternative technique with exceptional potential in various biomedical directions including the sterilization and sanitation of different surfaces [114,121,125], wound healing [124,126], tissue regeneration [124,127], treatment of infections [127,128], and cancer [80,81,82]. In particular, interactions of NTP with both Gram-positive and Gram-negative bacteria, biofilms, spores, and fungi have been extensively studied over the past decade [116,129,130]. A notable antibacterial efficiency of NTP has been reported against a substantial number of high-risk pathogens such as *Staphylococcus* spp., *Escherichia coli*, *Salmonella enterica*, *Pseudomonas aeruginosa*, and *Bacillus* spp. in the form of liquid bacterial suspensions as well as surface-grown biofilms [131,132,133,134,135].

Nevertheless, the exact mechanism of NTP interactions with prokaryotic or eukaryotic cells has not been fully understood, which is caused by the great diversity of the biological targets, chemical, and physical properties of NTP, and above all by a significant variability of active NTP components and their subsequent effects on the target [113]. As discussed earlier, the primary components of NTP are ROS, RNS, UV radiation, and electrical field, all of which possess antimicrobial properties. More specifically, ROS and RNS act as pro-oxidants on the outer cellular structures of bacteria [129], leading to lipid peroxidation, DNA fragmentation, protein alterations, and cell death in microorganisms [136]. Charged particles and electrostatic force may equally play an important role in disintegrating the outer cell membrane [137]. Furthermore, UV radiation affects the dimerization of thymine bases in DNA, leading to irreparable damage to the nucleotides [138]. Whilst all these effects are desirable in terms of bacterial eradication, it must be remembered that the NTP treatment of semen samples may exhibit a similar negative impact on the sperm cells. As such, for NTP to be successfully applied as an antibacterial treatment option for the processing of ejaculates, pivotal reports have recently emerged studying the effects NTP on male gametes.

In the experiments designed by Mazhir et al. [139], spermatozoa collected from patients suffering asthenozoospermia were exposed to NTP discharged from a plasma jet for 120 s. The samples were compared before and following plasma treatment. The authors concluded that NTP treatment led to improved sperm motility and a lower occurrence of spermatozoa with medium and high levels of DNA damage. Inversely, the DBD plasma treatment of normozoospermic semen samples was studied by Tvrdá et al. [140]. Exposure to NTP with a power input of 40 W for 15 s or 30 s had no negative effects on the sperm motility, membrane integrity, mitochondrial activity, or DNA stability. However, a prolonged NTP treatment of 60 or 90 s impaired all target sperm quality characteristics in a time-dependent manner. The authors suggest the probable mechanism of action of high NTP doses may be connected to ROS overproduction, leading to the destabilization of the plasma membrane, lipid peroxidation, and a loss of DNA integrity. Similar observations were published by Zhang et al. [141], who subjected rooster semen to DBD plasma of different intensities for 20 s. According to the study, DBD plasma treatment of 11.7 kV led to maximum sperm motility by controlling ROS levels, upregulating the expression levels of antioxidant genes, while boosting the release of adenosine triphosphate and the activity of mitochondrial respiratory enzymes. Nevertheless, a prolonged exposure to NTP or higher plasma voltage exerted significant adverse effects on the sperm vitality and oxidative profile. Summarizing the preliminary data from the currently published studies, it may be indicated that appropriate plasma doses and exposure times need to be carefully selected to preserve sperm vitality, should NTP be used in the practical management of bacteriospermia in the future. At the same time, studies on sperm-bacterial co-cultures must be designed and carried out in order to define the optimal NTP exposure conditions for the effective eradication of bacteria while preserving the desirable sperm structural integrity and functional activity.

## 6. Magnetic-Activated Cell Sorting

Magnetic-activated cell sorting (MACS) is a modern sperm separation technique that has been successfully employed to eliminate apoptotic spermatozoa that may be responsible for AI or ART failure even in normozoospermic males. The magnetic separation of semen samples has been shown to yield highly motile and more cryoresistant spermatozoa with desirable viability, morphology, and fertilization potential [142,143,144,145]. The principle of the technique lies in the utilization of colloidal super-paramagnetic microbeads of approximately 50 nm, composed of a biodegradable matrix that is conjugated with annexin V [145]. This phospholipid-binding protein selectively binds to the phosphatidylserine residue that is externalized on the cellular surface if the membrane is damaged and the cell has entered apoptotic death [146]. Hence, a positive annexin V signal indicates that the sperm membrane integrity has been compromised, and the sperm cell is highly unlikely to accomplish fertilization [147].

**Figure 2:**
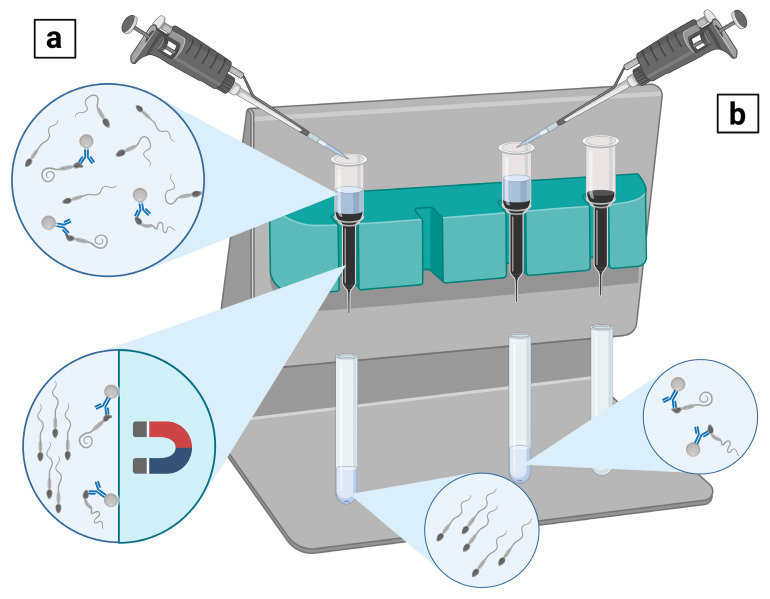
Magnetic-activated cell sorting (MACS) overview. (**a**) The separation columns are surrounded by a magnetic field and loaded with semen mixed with annexin V labeled microbeads. The magnetic field will retain apoptotic spermatozoa tagged to the microbeads while non-apoptotic spermatozoa will flow through the column. (**b**) Once non-apoptotic spermatozoa are collected, the column is flushed, and apoptotic spermatozoa are washed out.

The technique involves the incubation of the sperm suspension with annexin V-labelled microbeads at laboratory temperature for 15–20 min. The microbeads have no known effects on the sperm structure or function and, thanks to their biodegradability, it is not necessary to remove these following co-incubation [148]. The suspension is then loaded into separation columns placed within iron spheres on a stand surrounded by a magnetic field of 0.5–1.5 Tesla [100]. The resulting setting causes spermatozoa that are non-apoptotic to pass through the magnetic field, whereas sperm that are apoptotic, and thus labeled with the microbeads, are retained in the magnetic field [142,144]. Based on the labelling and binding process and subsequent magnetic separation, two sperm cell fractions will be obtained: an annexin-negative fraction, representing non-apoptotic and thus membrane-intact spermatozoa and an annexin-positive fraction, standing for apoptotic spermatozoa with an altered membrane. Following separation, the column will be removed from the MACS separator and the retained cells will be eluted with the annexin V binding buffer [148] (Figure 2).

As opposed to conventional separation methods stemming from sperm motility and/or density, MACS is based on sperm differentiation on a molecular level. The application of MACS separation in practical settings has been shown to yield highly motile spermatozoa [144] with a lower incidence of acrosomal, midpiece, and tail defects as well as cytoplasmic droplets [149,150]. Furthermore, MACS has been revealed to be highly effective in improving fertilization rates and oocyte penetration that are crucial pre-requisites for ARTs [151,152]. Consequently, MACS application has improved pregnancy rates in comparison to spermatozoa processed by density gradient alone [153]. The technique has been reported to be particularly helpful in optimizing the sperm freezing–thawing procedure by removing apoptotic spermatozoa prior to cryopreservation and hence improving the resulting post-thaw sperm cryosurvival, motility, and mitochondrial activity [148,154].

Whilst MACS is regarded as a fast, convenient, and reliable technique that is based on inexpensive and easy-to-use equipment, providing consistent results in terms of good cell purity and recovery, its major limitation currently lies in the removal of primary apoptotic spermatozoa. The technique is limited by its ability to remove other components of semen including seminal plasma, immature sperm, leukocytes, debris, and, eventually, bacteria. As such, MACS alone is not suitable for bacterial elimination, which is why it is recommended to combine the technique with other sperm-processing protocols so that these can complement each other and provide spermatozoa with the highest quality while at the same time leaving them free from any factors that may endanger their structural integrity or functional activity [144]. In fact, it has been reported that the combination of density gradient centrifugation and MACS is currently superior to other sperm preparation protocols in terms of removing immature spermatozoa, leukocytes, epithelial cells, and bacteria whilst eliminating already damaged spermatozoa with compromised membranes, activated apoptosis, oxidative damage, and DNA fragmentation [151,154].

## 7. Microfluidics and Microelectrophoresis

The most rapidly evolving field for sperm selection, preparation, and purification is based on microfluidics and microflow systems, considered as the semen processing methods of the 21st century [155]. The principle of microfluidics lies in the study and control of small volumes (picoliters or microliters) of fluids inside micrometer-sized channels [156] made from transparent polydimethylsiloxane silicon polymers that are nontoxic to the cells [157]. These channels are ideally suited to spermatozoa as these may be designed to resemble the architecture of the female reproductive tract, thus enabling a biomimicry-based sperm selection simulating an in vivo environment [155]. Different readily available lab-on-chip approaches may be used to purify and select spermatozoa based on motility [157,158], electrophoresis [159], flow [160], optical forces [161,162], chemical gradients [163] or chemotaxis [164,165].

Currently, the most popular devices take advantage of passive microfluidics which selects spermatozoa based on boundary-following behavior. One example uses two parallel laminar flow channels where immature and non-motile spermatozoa, leukocytes, bacteria, or debris will move along their initial stream and be collected from one outlet, while motile spermatozoa will swim into a parallel streamline and be recovered from a separate outlet [158]. Another device that has been designed for sperm processing consists of a radial network of channels containing a medium that simulates the viscosity of the female reproductive fluid, separating spermatozoa into straight, right, and left swimmers. Raw semen will be loaded into the device and kept undisturbed for 15 min. Motile spermatozoa will move through the microchannel end exit from one outlet, while immotile spermatozoa, debris, leukocytes, and bacteria will be retained in the inlet [166]. Whilst pioneer microfluid devices for semen processing relied on sperm selection based on their size or motility exclusively, a higher effectivity of the system has been achieved by incorporating chemo-attractants, such as cumulus cells, oviductal or fallopian tube cells [164,167].

Amongst the numerous advantages that come with microfluidics, the small sample volume, short processing times, increased automation, and the ability to work with single cells in a minimally invasive manner are the most prominent ones [168,169]. The yield of cells recovered by microfluid systems is comparable to other conventional methods [160], while the recovered spermatozoa present with a better progressive motility, morphology, and DNA integrity [170]. As such, microfluid platforms present with a great potential, particularly in the field of semen processing for the purposes of in vitro fertilization (IVF) and intracytoplasmic sperm injection (ICSI) in humans [170,171,172] as well as animals [173,174]. Keeping bacteria as a notable component of semen, Jeon et al. [175] have just recently proposed a fully automated multi-dimensional double spiral (MDDS) microfluidic device that can effectively isolate spermatozoa from other non-sperm seminal cells (including bacteria) in raw semen samples without any pre-washing steps. Preliminary data have revealed the excellent performance of the MDDS platform with ~80% of sperm cell recovery, and >99.95% removal of 10 μm beads serving as a surrogate for leukocytes or bacteria from both low-viscosity as well as high-viscosity ejaculates. As such, this microfluidic device could become an efficient and automated tool to collect purified sperm suspensions in the absence of any pro-inflammatory leukocytes or contaminating bacteria as an alternative to swim-up or density centrifugation, which often suffer from low sperm recovery and labor-intensive intermediate steps in the laboratory.

**Figure 3:**
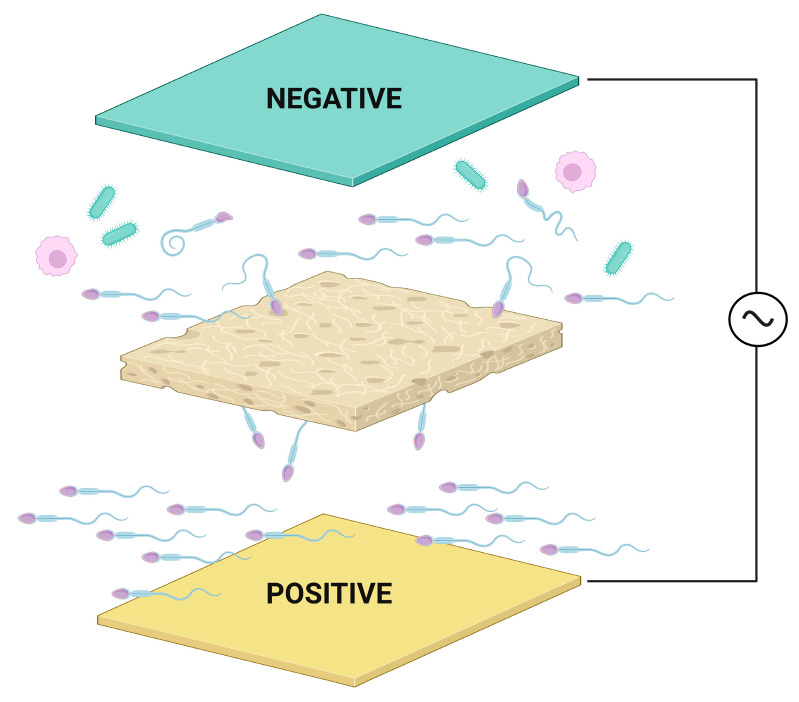
Sperm microelectrophoresis overview. Heathy and mature sperm are charged negatively and under electric current will migrate to the anode (positive electrode) and will be separated from immature or damaged sperm. At the same time, spermatozoa are small enough to pass through a polycarbonate membrane and will be separated from other cell types present in semen.

Finally, microelectrophoretic techniques retrieve spermatozoa from semen based on their charge and size [160,176,177]. The charge of mature spermatozoa is inherently negative [160,178], which develops with sialic acid formation on the sperm surface late in the maturation process. As such, within an electric field, more mature and negatively charged spermatozoa will be drawn towards the positively charged anode [177]. This process will enable the separation of mature and viable spermatozoa from their damaged or immature counterparts. At the same time, spermatozoa will be separated from other seminal components based on their size [160,177]. During microelectrophoresis, a polycarbonate membrane separates the electrophoretic chamber with pores large enough to enable vital spermatozoa to pass through while other larger undesirable cells in the semen will be retained on the membrane (Figure 3). A microelectrophoretic device called Felix™ that separates high quality sperm from raw semen, by a process combining electrophoresis and size exclusion membranes, is already commercially available. The device consists of a system of chambers connected with platinum-coated titanium electrodes. The sample and harvest chamber are separated by a 5 μm thick polycarbonate membrane which filters out vital and healthy spermatozoa from other contaminating cells such as germ cells, leukocytes, and, eventually, bacteria. One separation cycle lasts 6 min with a constant current of 75 mAmps and a variable voltage of 18–21 mV [160]. The device has been already tested out against conventional density gradient centrifugation across five international ART centers with both methods being capable of isolating highly vital and motile spermatozoa. Whilst the yield was lower with the Felix™ device, the recovered spermatozoa presented with a significantly improved DNA integrity relative to density gradient centrifugation. More importantly, the preparation time and workflow were significantly reduced with microelectrophoresis which may be a highly welcome asset in clinical andrology [179].

## 8. Conclusions

In summary, we have described selected strategies that are or may be used for the eradication of bacteria from semen while at the same time yielding spermatozoa that present with desirable viability and fertilization potential. Nevertheless, each ejaculate should be evaluated beforehand to determine the best method for bacterial decontamination depending on the initial state of the sample and its use. Future research is necessary to improve the efficiency and safety of each approach. Novel strategies to prevent bacteriospermia may improve the probability of obtaining structurally intact, viable, and mature spermatozoa without the necessity for antibiotic supplementation to semen extenders and/or ART media; nevertheless, these will still need large-scale and multicenter optimization and standardization trials before their incorporation into everyday andrological practice.

## Figures and Tables

**Table 1:** A summary of antibiotic resistance profiles of bacteria recovered from human and animal semen.

Subjects	Sample	Outcomes	Ref.
Men with or without urogenital inflammation	Fresh semen	Out of the isolated Gram-positive microorganisms, 33.7% were resistant to tetracycline and 46.9% were resistant to sulfamethoxazole-trimethoprim. In the case of Gram-negative microorganisms, 51.5% were resistant to ampicillin, 43.5% to piperacillin, 25.0% to amoxicillin-clavulanic acid, 23.4% to ciprofloxacin, and 20.3% to levofloxacin.	[48]
Fertile and infertile men	Fresh semen	78.9% *Escherichia coli* isolates were resistant to tetracycline, 73.6% to levofloxacin, 57.8% to tobramycin or chloramphenicol. A total of 66.6% of *Klebsiella pneumoniae* isolates were resistant to gentamicin, tobramycin, tetracycline, and cefotaxime. A total of 80.0% *Staphylococcus epidermidis* isolates were resistant to gentamycin and 60% to streptomycin.	[49]
Normozoospermic men	Fresh semen	A total of 35.0% *Enterococcus faecalis* isolates were resistant to ampicillin. A total of 75.0% Staphylococcus *haemolyticus* isolates were resistant to tobramycin or tetracycline. A total of 70.0% *Staphylococcus hominis* and 32.0% *Staphylococcus capitis* isolates were resistant to tobramycin. A total of 68.0% *Staphylococcus epidermidis* were resistant to tetracycline.	[13]
Men with primary infertility	Fresh semen	High resistance of Gram-positive bacteria to rifampicin (48.0% isolates), clindamycin (40.0% isolates), and vancomycin (44.0% isolates). High resistance of Gram-negative bacteria to ceftazidime (20.0% isolates) and cefuroxime (40.0% isolates).	[50]
Infertile men with urogenital inflammation	Fresh semen	High *Staphylococcus aureus* resistance to ceftazidime and ceftriaxone (53.9% isolates), fluoroquinolone (69.3% isolates), cefotaxime (42.3% isolates), and oxacixicillin (61.5% isolates). High *Escherichia coli* resistance to ceftazidime (55.6% isolates), lindamycin and cefotaxime (66.7% isolates), and oxacixicillin (77.8% isolates). High *Klebsiella* spp. resistance to ceftriaxone (60.0% isolates) and oxacixicillin (90.0% isolates). High *Pseudomonas aeruginosa* resistance to ceftazidime, ofloxacine, and oxacixicillin (83.3% isolates), gentamycin, vancomycin, and cefotaxime (66.7% isolates).	[51]
Men undergoing fertility evaluation	Fresh semen	A rising trend for streptococci resistance to erythromycin. A rising trend for staphylococci to penicillin and ampicillin/sulbactam.	[52]
Bovine studs	Fresh, cooled, and packed semen	A total of 23.6% out of the isolated microorganisms presented with resistance to gentamicin, while 92.7% were resistant to streptomycin. Resistance to lincomycin/spectinomycin was observed for more than 23.6% of all isolates. A total of 22% of all isolates were resistant to all tested antibiotics.	[53]
Yearling bulls	Fresh semen	All *Serratia liquefaciens* and *Serratia quinivorans* isolates were resistant to cefazolin. All *Cronobacter sakazakii*, *Escherichia fergusonii*, and *Shigella boydii* were resistant to tetracycline. 2 *Shigella* and 1 *Salmonella* isolates were resistant to cefazolin, amikacin, and gentamicin.	[54]
Breeding rams	Fresh semen	One *Staphylococcus equorum* and one *Staphylococcus vitulinus* isolate were resistant to ciprofloxacin.	[15]
Breeding rams	Fresh and diluted semen	A total of 53% of all isolates were resistant to penicillin, 47% to erythromycin, 33% to oxytetracycline, 20% to ampicillin, 15% to streptomycin, 15% to co-trimoxazole, 13% to polymixin B, and 4% to spectinomycin.	[55]
Duroc boars	Diluted semen	All *Enterococcus hirae*, *Staphylococcus aureus*, as well as 50% of *Staphylococcus simulans* and *Staphylococcus chromogenes* isolates, were resistant to tigecycline. All *Acinetobacter iwoffi*, *Pseudomonas aeruginosa*, and *Pseudomonas putida* isolates (100%) were resistant to ciprofloxacin.	[32]
Breeding boars	Fresh semen	A total of 56.5% out of the isolated microorganisms presented with resistance to gentamicin and penicillin, while 58.7% were resistant to neomycin. Resistance to ceftiofur and lincomycin was observed for more than 47.8% of all isolates. A total of 50.0% of all isolates were resistant to ampicillin.	[56]
Breeding boars	Fresh and diluted semen	*Escherichia coli* isolates showed 50% resistance to amikacin and 70% resistance to gentamicin; *Staphylococcus epidermidis* was 12.5% resistant to amikacin and 50% to gentamicin; *Serratia marcescens* showed a resistance of 66.6% to amikacin and 50% to gentamicin; *Proteus* spp. isolates were 25% resistant to amikacin and 50% to gentamicin; *Streptococcus* spp. isolates were 50% resistant to gentamicin; *Staphylococcus aureus* isolates were 100% resistant to gentamicin.	[57]
Big 6 turkeys	Fresh semen	All *Staphylococcus lentus* isolates were resistant to chloramphenicol, linezolid, and tigecycline. *Enterococcus faecium* isolates showed resistance against imipenem, while ertapenem was shown to be ineffective against *Escherichia coli* and *Vagococcus fluvialis* isolates.	[16]
Breeding turkeys	Fresh and stored semen	All *Enterococcus faecalis*, *Bacillus subtilis* and corynebacteriaisolates were resistant to ampicillin-cloxacillin, cefuroxime, amoxicillin, and ceftriaxone. All *Escherichia coli* isolates were resistant to co-trimoxazole, ofloxacin, and nalidixic acid.	[58]
Lohmann Brown and ROSS 308 roosters	Fresh semen	*Citrobacter braakii* (75% isolates), *Enterococcus faecalis* (25% isolates), *Escherichia coli* (46% isolates), and *Staphylococcus epidermidis* (20% isolates) were resistant to ampicillin. Resistance to tetracycline was observed in *Staphylococcus epidermidis* isolates (60%), while 36% *Escherichia coli* isolates were resistant to chloramphenicol. Multiresistance patterns against several antibiotics were recorded, particularly in the cases of *Citrobacter braakii*, *Enterococcus faecalis*, *Escherichia coli*, and *Staphylococcus epidermidis*.	[17]
Broilers, layers, and fattening turkeys	Fresh semen	High resistance rates were identified in *Enterococcus faecalis* and *Enterococcus faecium* for lincomycin (72–99% isolates) and tetracycline (67–82% isolates). More than half of *Enterococcus* isolates were resistant to gentamicin (54–72% isolates), erythromycin (44–61% isolates), and tylosin (44–56% isolates). At total of 89 out of 145 *Enterococcus* isolates were resistant to three or more antimicrobial classes.	[59]
Indian red jungle fowl	Cryopreserved semen	*Escherichia coli* showed high resistance towards streptomycin. *Staphylococcus* spp. isolates were resistant to kanamycin. *Bacillus* spp. isolates were resistant to neomycin and streptomycin	[60]

## Data Availability

Data collected for the purposes of this paper are available upon reasonable request from the corresponding author.
